# Food Neophobia and Avoidant/Restrictive Food Intake among Adults and Related Factors

**DOI:** 10.3390/nu16172952

**Published:** 2024-09-02

**Authors:** Agnieszka Białek-Dratwa, Wiktoria Staśkiewicz-Bartecka, Agata Kiciak, Aleksandra Wardyniec, Mateusz Grajek, Şule Aktaç, Zehra Margot Çelik, Güleren Sabuncular, Ayşe Hümeyra İslamoğlu, Oskar Kowalski

**Affiliations:** 1 Department of Human Nutrition, Department of Dietetics, Faculty of Public Health in Bytom, Medical University of Silesia in Katowice, ul. Jordana 19, 41-808 Zabrze, Poland; 2 Department of Food Technology and Quality Assessment, School of Public Health in Bytom, Medical University of Silesia in Katowice, ul. Jordana 19, 41-808 Zabrze, Poland; 3 Department of Public Health, School of Public Health in Bytom, Medical University of Silesia in Katowice, ul. Piekarska 18, 41-902 Bytom, Poland; 4 Department of Nutrition and Dietetics, Faculty of Health Sciences, Marmara University, Istanbul 34722, Turkey; sule.aktac@marmara.edu.tr (Ş.A.); zcelik@marmara.edu.tr (Z.M.Ç.);

**Keywords:** appetite, fear, picky eating, eating behavior, eating disorder, ARFID, food neophobia

## Abstract

Avoidant/restrictive food intake disorder (ARFID) includes age-inappropriate feeding behaviors in eating patterns, including food neophobia, defined as refusal or reluctance to eat new or unknown foods. This study aimed to assess the prevalence of ARFID and food neophobia among adults and determine the related characteristics of these risks. The study used an anonymous survey questionnaire consisting of three parts as the research tool. The first part of the questionnaire was a metric and concerned socio-demographic data. The Food Neophobia Scale (FNS) and the Nine-Item Avoidance/Restrictive Food Disorder Screen Questionnaire (NIAS) were used to evaluate the eating disorders. The survey included 309 people (60.2% women, 39.8% men) aged 18–77 years. NIAS results indicated that 15.2% of the subjects showed food selectivity, and 11.0% had food anxiety. In the FNS assessment, 42.4% had a low risk of food neophobia, 38.2% a medium risk, and 19.4% a high risk. A higher risk of food neophobia correlated with higher NIAS scores, indicating a higher risk of ARFID (*p* = 0.00231). The NIAS score increased with the risk of food neophobia (*p* = 0.000). Respondents at low risk of neophobia were most likely to avoid several products (83.97%), while in the high-risk group, 56.67% did not want to eat a favorite food enriched with a new ingredient. A higher risk of neophobia was correlated with more food avoidance and adverse reactions to new foods (*p* = 0.000). A higher risk of food neophobia is strongly correlated with a higher risk of ARFID. Although demographics did not significantly impact NIAS results, some trends were noted, such as higher scores among older and underweight people. Those with a higher risk of food neophobia show more food avoidance and a greater reluctance to experiment with new ingredients. Public education should emphasize that eating disorders affect both sexes equally, with tailored interventions for high-risk groups such as the elderly, rural populations, and those with lower education. Health policies should promote access to nutrition education, psychological support, and diverse food options, while further research is needed to improve targeted interventions.

## 1. Introduction

Eating disorders are psychological disorders that can affect the physical, psychological, and social functions of individuals to different degrees and can lead to severe outcomes [1]; disorders can occur in individuals of any gender, age, ethnicity, body shape, or body weight [2]. While eating disorders such as anorexia nervosa or bulimia nervosa are related to body weight and body perception, some eating disorders are found to be related to loss of interest in food or eating and avoidance of sensory features of food such as smell and appearance [1,3,4,5].

Food neophobia has been defined as a reluctance to try unfamiliar foods [6]. This eating behavior is an inherited tendency passed down from generation to generation whereby some individuals are overly picky about foods, possibly to avoid the toxicity of an unknown food source [7]. This situation leads to decreased fruit and vegetable consumption, poor dietary diversity, and poor adult diet quality [6,8]. The avoidance of new foods seen in food neophobia has been associated with metabolic risk factors and an increased risk of disease outcomes [9,10].

Avoidant/restrictive food intake disorder (ARFID) was defined as an eating disorder for children aged 6 years and younger in the Diagnostic and Statistical Manual of Mental Disorders-IV (DSM-IV) criteria. However, according to DSM-5 criteria published in 2013, the age restriction was removed. It was defined as an eating disorder that can be diagnosed in children, adolescents, and adults [1]. In DSM-5, it was stated that patients were not afraid of weight loss but could not meet their nutritional needs for various reasons after the exclusion of chronic illness, mental illness, or inability to access food [4]. ARFID includes age-inappropriate feeding behaviors in eating patterns, including food neophobia, which is defined as refusal or reluctance to eat new or unknown foods. While picky eating behaviors are considered normal in early childhood, they are expected to decrease with age [4]. In ARFID, food intake is restricted due to avoidance of sensory properties of food (e.g., taste, smell, and texture), lack of interest in food or eating, or feared adverse food-related events (e.g., choking and vomiting) [11,12].

ARFID is characterized by a persistent inability to meet appropriate nutritional and energy needs [5]. ARFID may lead to weight loss, failure to gain weight, nutritional deficiency, dependence on nutritional supplements or enteral nutrition, and psychosocial problems [13]. Particularly, selective eating behaviors may be associated with specific nutritional deficiencies, while total energy intake may be adequate and not impact weight. Deficiencies of various vitamins and minerals and related complications have been documented in patients undergoing treatment for ARFID [14]. A negative correlation was also identified between food neophobia, nutritional intake, and healthy eating [15]. There are numerous similarities between the behavioral expressions of food neophobia and ARFID [16]. The intensity of ARFID symptoms was found to be positively correlated with food neophobia in patients [17].

ARFID is diagnosed based on medical and psychological assessment [11]. Although the diagnostic criteria for ARFID in the DSM-V include four basic guidelines [1,18], these often need to be revised. Additional diagnostic tools have been developed, such as the PARDI-AR-Q, EDY-Q, and NIAS [11,18,19,20,21]. The ARFID diagnostic criteria include the following guidelines: (A) the eating disorder is associated with weight loss, nutrient deficiency, need for enteral feeding or significant changes in psychosocial functioning, (B) the disorder is not the result of food inaccessibility, cultural or religious beliefs, (C) the disorder is not associated with other eating disorders, and (D) the disorder is not dependent on medical conditions or comorbidities [1].

The PARDI-AR-Q is a 32-item interview that assesses four critical features of ARFID, such as the severity of the disorder, sensory sensitivity, fear of food, and lack of interest in food [11,19]. The EDY-Q, on the other hand, is a self-report questionnaire with 14 items, 12 of which focus on ARFID, based on traits from the DSM-V [20]. The NIAS is a nine-item questionnaire to assess pickiness of eating, lack of appetite, and fear of food [19]. Untreated ARFID leads to cachexia, food restriction, and nutritional deficiencies, which can result in serious health consequences [18,21].

Treatment of ARFID remains a challenge, as there is a lack of specific validated therapeutic approaches [5,11]. Treatment should be multidisciplinary, involving medical, psychiatric, and dietary care, with the possibility of hospitalization in severe cases [11,18]. Medical management consists of assessing the patient’s condition and implementing appropriate outpatient treatment or, if necessary, hospitalization [22,23]. Within psychological management, cognitive–behavioral therapy (CBT-AR) is particularly effective in treating patients over 10 years of age with ARFID [24]. Although there are no approved medications for ARFID, psychotropic drugs such as lorazepam and mirtazapine are used in some cases to alleviate symptoms of the disorder [11].

The diagnosis of food neophobia is most often based on the Food Neophobia Scale (FNS), which assesses the level of food neophobia [25]. Other tools for assessing different aspects of this disorder include VARSEEK, FAS, FEQ, DSI, and FNTS [26]. Treatment of feeding neophobia in children is usually not necessary, but psycho-dietary therapy is recommended in cases that require intervention [27,28].

Both food neophobia and ARFID are eating disorders that involve avoidance or restriction of food intake. Although both disorders can lead to serious health problems, ARFID usually requires more extensive medical management [11]. Untreated eating disorders, such as eating neophobia and ARFID, can lead to serious health problems, including malnutrition and diet-related diseases [5,11,29].

A scoping review shows that most of the studies in this field have focused on child and adolescent populations, while few studies have been conducted with adults [13].

This study aimed to assess the prevalence of eating disorders such as ARFID and eating neophobia among adults and determine the prevalence of ARFID and food neophobia by gender, age, education, and place of residence. The secondary aim of the study was to investigate the selectability of food groups and foods among participants.

## 2. Materials and Methods

### 2.1. Study Design and Population

The study was conducted from February 2023 to May 2023 in Poland. The respondents were persons with Polish citizenship. The research process used a recruitment method based on the snowball technique, where each participant was asked to pass the questionnaire to the next potential respondents. Participation in the survey was not only completely anonymous but also entirely voluntary, respecting the autonomy of each participant. The total group of respondents consisted of 309 people.

The study’s inclusion criteria were adults over 18 years of age, correctly and reliably completed, and no disease that determines a specific diet eliminating various ingredients/food products.

The exclusion criteria were an incomplete questionnaire and a questionnaire completed unreliably. To verify the reliability of the questionnaire completion, 3 control questions placed in different parts of the questionnaire were used. Twelve unreliably completed questionnaires were excluded from the study. Those suffering from diseases affecting their nutrient intake, such as anorexia, bulimia, active cancer, cancer treatment, or other serious diseases, were excluded from the study.

The Declaration of Helsinki and the Act on the Profession of Physicians and Dentists conducted the study. The Bioethics Committee of the Medical University of Silesia in Katowice evaluated and approved the study protocol (PCN/0022/KB/68/I/20).

### 2.2. Data Collection and Measurements

The questionnaire was designed and implemented using the Google Forms platform (Google LLC, Mountain View, CA, USA). The survey was implemented using the questionnaire method, using an indirect survey technique through computer-assisted web interviewing (CAWI). Participants completed the questionnaire electronically.

The anonymized questionnaire used to obtain data for the study consisted of 4 parts: a metric, a standardized Food Neophobia Scale (FNS) questionnaire [30], a standardized Nine-Item Avoidance/Restrictive Food Disorder Screen (NIAS) [19] questionnaire, and follow-up questions. The questionnaire consisted of 44 questions. The metrics section included questions about participants’ gender, age, place of residence, education, occupational status, and prevalent diseases. In addition, variables used to generate BMI (body mass index) data, such as body weight and height, were also provided.

### 2.3. The FNS Questionnaire

The FNS (Food Neophobia Scale), a 7-item survey validated by Pilner and Hobden, is the most often used scale to assess food neophobia in adults [30]. The FNS questionnaire used to assess the level of food neophobia includes ten statements covering both neophobic and neophilic behaviors. For each statement, the participant had to mark one of seven available responses with which they most identified. It contains 10 statements, 5 of which refer to neophobic behavior and the remaining five to neophilic behavior. In line with the FNS methodology, reverse scoring was applied to 5 of the 10 statements made. (1) I try new and different types of food all the time. (2) I like food from different countries. (3) When I am away from home, I try new types of food. (4) I eat almost everything. (5) I would like to eat food from other regions of Poland or other countries. When answering the questions, the participant chooses one of the seven possible answers (1—strongly disagree; 7—strongly agree) with which they most identify. The higher the FNS, the stronger the food neophobia. It is categorized into 3 groups according to FNS scoring. Less than 27 indicates low risk, 28–40 indicates average risk, and greater than 40 indicates high risk [31]. For the general identification of potential ARFID cases, a positive screening score on any of the NIAS subscales (≥14 NIAS-avoidant eating, ≥13 NIAS-appetite, and ≥14 NIAS-fear) is recommended.

### 2.4. The NIAS Questionnaire

The NIAS questionnaire, a comprehensive tool, was used to assess the avoidant/restrictive eating patterns of participants. It consisted of a total of nine questions that addressed issues such as sensory aversion toward food, level of disinterest in food, and fear of consequences associated with eating a particular food. As with the FNS questionnaire, the participants had to tick one of seven responses that they identified next to each question. This thorough approach ensures that we understand the full spectrum of your eating patterns.

Each of the 3 questions in the subscales is scored from 0 to 7 points, with the individual scales ranging from 0 to 21, with higher scores indicating higher levels of each indicator (picky eating, disinterest, and fear). All items can also be summed to calculate a total score ranging from 0 to 63, with higher scores indicating higher levels of ARFID. For the general identification of potential ARFID cases, a positive screening score on any of the NIAS subscales (≥14 NIAS-avoidant eating, ≥13 NIAS-appetite, and ≥14 NIAS-fear) is recommended [19].

The final part of the survey consisted of follow-up questions. These dealt with issues such as limits on the number of products and foods eaten and the reasons for not eating them, an assessment of the desire to try a disliked product in a different form, eating a dish made by a third party, an assessment of discomfort caused by eating in restaurants, the tendency to eat more variety or to compare the size of the food eaten to other people, the types of diets followed, and the presence of diseases or food allergies. The survey also included questions relating to household nutrition and potentially resulting dietary restrictions.

### 2.5. Statistical Analysis

For statistical analysis, we used statistical tests, which were performed in Statistica v. 13.3 (StatSoft Inc., Tulsa, OK, USA). Descriptive statistics in the data evaluation include numbers and percentages for qualitative variables and mean, standard deviation, median, minimum, and maximum for quantitative variables. The Kolmogorov–Smirnov test checked the variables’ conformity to a normal distribution.

Data such as gender, age, occupation, place of residence, BMI, FNS, FNS interpretation, and NIAS did not have a normal distribution; therefore, non-parametric tests were used further. The Mann–Whitney U test was used to compare mean NIAS values. The non-parametric Chi2 test was used to analyze qualitative data. Results were assessed at the 95% confidence interval, and the level of statistical significance was set at *p* < 0.05.

## 3. Results

### 3.1. Characteristics of the Study Group

A total of 309 people took part in the study, aged 18–77 years. The most significant respondents were women, 60.2% (n = 186), while the proportion of men was 9.2% (n = 123). Among the respondents, the largest age group comprised respondents aged 18–30 years, 45.3% (n = 140), followed by respondents aged 31–43 years, 24.6% (n = 76), and respondents aged 44–56 years, 20.7% (n = 64). Respondents in the 57–69 age group accounted for 7.1% (n = 22) of all respondents. The smallest group were those aged 70–77 years, 2.3% (n = 7). More than half of the respondents, 52.4% (n = 162), indicated a city of more than 100,000 inhabitants as their place of residence. Overall, 25.2% (n = 78) of respondents chose a city of 20,000 and 100,000 inhabitants as their residence, followed by 13.0% (n = 40) selecting a city of less than 20,000 inhabitants. However, only 9.4% (n = 29) of respondents described their residence as a village. Among the respondents, as many as 40.8% (n = 126) were people with secondary education. Overall, 17.5% (n = 54) of respondents declared an incomplete tertiary education, while tertiary education was declared by 28.2% (n = 87) of respondents. Those with vocational education accounted for 13.3% (n = 41) of all respondents. Primary education was marked by only 0.3% (n = 1) of the respondents. When asked about their occupational status, as many as 60.8% (n = 188) of respondents selected the answer “working”, 17.5% (n = 54) of the respondents declared their status as “student”, followed by “working student”, 9.7% (n = 30), and unemployed, 2.9% (n = 9) (Table 1).

### 3.2. Results Concerning the NIAS and FNS Questionnaires

Based on the data collected from the NIAS questionnaire, 15.2% (n = 47) show the occurrence of picky eating and the same percentage of respondents show a lack of interest in food. On the other hand, 11.0% (n = 34) of respondents present fear and apprehension toward food in their response to the questionnaire. Considering the prevalence of food neophobia risk according to the FNS questionnaire, 131 people (42.39%) have a low risk of food neophobia, 118 people (38.18%) have a medium risk of food neophobia, and 60 people (19.41%) have a high risk of food neophobia (Table 2).

Table 3 shows the FNS risk of food neophobia and the number of NIAS subscales indicative of ARFID. By analyzing the risk of food neophobia according to the FNS and the scores of the NIAS subscales indicative of ARFID in the subscales picky eating, appetite, and fear, the higher the risk of food neophobia, the more subscales whose score indicates the risk of ARFID (*p* = 0.00231). The 14 individuals with 3 subscales of ARFID with a positive score had a mean NIAS questionnaire score of mean value Me = 49.5.

Among the entire group of respondents, the mean of the ARFID hazard index was 25.4 ± 9.3. When broken down by gender, there is little difference in the results of the mean value: women 25.5 ± 10.0 and men 25.1 ± 8.2. Underweight respondents show the highest risk of developing an eating disorder among all respondents, 30.7 ± 6.5, compared to average weight (24.9 ± 10.2), overweight (25.2 ± 8.6), and obese (25.2 ± 7.9) respondents, but statistical analysis shows that the differences are not statistically significant (*p* = 0.776814). Respondents between 57 and 69 years of age had higher ARFID scores than the rest of the respondents, 29.5 ± 9.3. Also high were those between 70 and 77 years of age, 27.3 ± 5.9, and 44 and 56 years of age, 27.1 ± 10.2. The rest of the respondents had similar results, but statistical analysis indicates that the differences are not statistically significant (*p* = 0.147502). According to the analysis of the NIAS questionnaire, survey participants living in rural areas have the highest risk of ARFID, 29.1 ± 10.5. Respondents living in a city of less than 20,000 and in a city of 20,000 to 100,000 obtained similar results about each other (Table 4).

In contrast, survey participants living in a city with more than 100,000 inhabitants showed the lowest risk of ARFID, but statistical analysis shows that the differences are not statistically significant (0.739512). Among the respondents, the highest average ARFID risk score was shown by those with vocational education, 29.3 ± 8.1, and those with primary education, 29 ± 0. Respondents with secondary and incomplete university education received scores of 25.4 ± 8.9 and 25.5 ± 9.7, respectively. Respondents with tertiary education received the lowest NIAS scores. The result is not statistically significant (*p* = 0.235516)

In contrast, a statistically significant increase in mean NIAS score and risk of food neophobia was observed. The higher the risk of food neophobia, the higher the final NIAS value (*p* = 0.000). The mean NIAS for those at low risk of neophobia was 19.7 ± 5.7; for those at medium risk of neophobia, 25.8 ± 6.7, and for those at high risk, 36.6 ± 9.5.

The study’s results showed variation in the risk of food neophobia by gender, BMI, age, place of residence, education, and occupational status. Women had a significantly higher mean FNS score than men (*p* = 0.019). Regarding BMI, the most significant differences were observed in the underweight group, which had the highest mean FNS score (*p* = 0.404, no statistical significance). Age analysis showed that those aged 57–69 had the highest mean FNS score, while the lowest mean score was observed in the 18–30 years group (*p* = 0.338, no statistical significance). Place of residence showed no significant differences in FNS scores, although rural residents had a slightly higher mean score than urban residents (*p* = 0.651, no statistical significance). In terms of education, those with primary education had the highest mean FNS score, while the lowest was found among those with a bachelor’s degree (*p* = 0.774, no statistical significance). Regarding occupational status, unemployed people and pensioners had the highest mean FNS score, while the lowest was recorded among working pensioners (*p* = 0.563, no statistical significance) (Table 5).

The study analyzed the relationship between the level of risk of food neophobia and various aspects of eating behavior. Those with a low risk of neophobia were most likely to declare that they do not eat only a few foods (83.97%). This percentage decreased as the risk of neophobia increased: 66.10% for medium risk and 58.33% for high risk. For those who do not eat a dozen or more products, the percentages were higher in the groups with a higher risk of neophobia. A higher risk of food neophobia is associated with a higher number of foods that the respondents avoid. These differences are statistically significant (*p* = 0.0325) (Table 6).

In the low-risk food neophobia group, 93.13% were willing to eat a dish prepared by a third party, compared to 88.98% in the medium-risk group and only 61.67% in the high-risk group. The percentage of people who were not willing to eat such a dish increased with the risk of neophobia, reaching 35.00% in the high-risk group. A higher risk of food neophobia is associated with a lower willingness to eat food prepared by a third party. These differences are statistically significant (*p* = 0.0063).

Those at low risk of food neophobia were the most likely to eat a favorite food enriched with a new ingredient (92.37%), while in the high-risk group, only 38.33% would agree. The percentage of people refusing increased significantly with the risk of neophobia, reaching 56.67% in the high-risk group. A higher risk of food neophobia correlates with a greater reluctance to experiment with new ingredients in familiar foods. These differences are statistically significant (*p* = 0.000).

In the low-risk food neophobia group, 77.10 percent of respondents declared that they had no problem eating new foods, while only 13.33 percent were in the high-risk group. Feelings such as uncertainty, disgust, anxiety, fear, and stress were more common in the high-risk group. A higher risk of food neophobia is associated with more negative feelings toward eating new foods. These differences are statistically significant (*p* = 0.000).

## 4. Discussion

Several studies have investigated food neophobia seen in childhood and adolescence and its association with other nutritional behaviors and overall health but relatively little information exists about the food neophobia seen in adults and its interplay with socio-demographic characteristics and nutritional aspects. The present study explored the relationship between food neophobia and the presence of ARFID and sociodemographic factors, including age, gender, BMI, place of residence, and educational and occupational status, which are known to have considerable impacts on food selection, nutritional habits, and behaviors.

In our study, no significant relationship was found between ARFID/food neophobia and the socio-demographic characteristics of participants such as age, BMI, place of residence, or educational and occupational status. Women had a significantly higher risk of food neophobia than men. It is emphasized in the literature that food neophobia increases with increasing age [31,32,33,34]. In our study, the risk of developing food neophobia is most pronounced between the ages of 57–77 years. It is likely that the study by Hazley et al. shows an increase in food neophobia in the age range of 54 to 64 years [27]. Similar findings were obtained during a study by Predieri et al. for the age group 46–60 years [35]. On the other hand, a study by Tian and Chen on the prevalence of food neophobia among students between the ages of 16 and 22 showed that the level of prevalence of this disorder was high, regardless of gender distinction [36].

Considering the effect of gender on food neophobia, conflicting results are found in the literature. Some studies involving adults show little or no effect of gender on food neophobia [37] while other studies show a weak effect of gender, with men generally found to be more neophobic than women [32,33].

Many studies found an association between high BMI and food neophobia, which leads to lower diet quality [15,38,39]. Even if it was not statistically significant, we found that the higher the BMIs get, the more food ARFID/food neophobia is seen.

Place of residence is another variable that might have an effect on food consumption and preferences. In accordance with our findings, Meiselman et al. [31] and Tuorila et al. [32] reported that food neophobia declines with urbanization, since people from rural areas may have fewer opportunities to be exposed to new and unusual foods. Likewise, the study by Hazley et al. shows an increase in food neophobia in people living in rural residences [27,39].

In a study conducted by Szakaly et al. in Hungary, a high level of neophobia was found in those with secondary or higher education [25]. Improbably, Jezewska-Zychowicz et al. found that people who have food neophobia had a lower level of education compared to people who do not have neophobia [40]. Similarly, in our study, the low level of education and the occupational status of ‘unemployed’ and ‘pensioner’ in the study indicate that both criteria increase the risk of food neophobia.

The study by Jezewska-Zychowicz et al. found that BMI did not influence the level of neophobia prevalence. Only the fear of side effects after consumption of certain products was noted, which was also observed in this study [40].

A study by Knaapil et al. highlighted that adults with nutritional neophobia tend to limit vegetables in their diet, and this tendency has a close link with childhood dietary restrictions [37]. In turn, the results of a study by Jeżewska et al. showed that in the Polish population, people with neophobia were more likely to consume foods such as meat and vegetables. The study also highlighted that the quality of meats and offal preferred by neophobic was significantly lower than among non-neophobic individuals, while vegetables and fruit were higher [40]. On the other hand, a study by Guzek et al. showed a relationship between food neophobia and restriction of fish and seafood—people with food neophobia avoided foods containing these particular ingredients. Also, they would not try a dish with an unattractive appearance and uninviting smell [41].

ARFID and adult eating neophobia are complex disorders that are influenced by many factors, including genetics, past experiences, and sensory and psychological factors. Understanding these factors is critical to developing effective treatment strategies and support for people with these disorders. Research suggests that traumatic experiences, such as choking or vomiting, may be linked to ARFID. These experiences lead to avoidance of food intake due to fears of possible repetition [42]. People with sensory hypersensitivity often avoid certain foods because of their texture, taste, or smell, particularly in people with ARFID [43]. ARFID is often associated with other disorders, such as anxiety, depression, or autism spectrum disorders [44]. Other studies indicate a link between genetic predisposition and the development of ARFID, suggesting the heritability of traits associated with the disorder [45]. Individuals with low self-esteem may develop ARFID as a mechanism to control their lives [46]. Previous experiences with restrictive diets may lead to ARFID, significantly if these were associated with guilt [47].

Early experiences with food can influence later attitudes toward food and a lack of variety in children’s diets can lead to neophobia in adulthood [48]. Fear of the unknown and the need for predictability may contribute to avoiding new foods [30,49]. Individuals with low self-esteem may avoid new foods for fear of social judgment [50]. Sensory hypersensitivity, as with ARFID, can lead to food neophobia [51].

The genetic determinants of food neophobia are an area of research that has gained prominence in recent years. Several studies suggest that food neophobia may be inherited to some extent and that genetics play a role in shaping our food preferences. A study of 5398 pairs of twins showed that food neophobia has a strong genetic basis. Data analysis showed that approximately 78% of the variation in behavior associated with food neophobia could be attributed to genetic factors, while 22% was related to environmental factors [52]. Taste preferences may be related to food neophobia, and genes may influence tastes we find appealing. A study of young adult twins showed that genetics plays a significant role in shaping food neophobia. The results strongly correlate neophobia taste preferences and personality traits [15]. Food neophobia has a significant genetic basis, which has been confirmed in various studies, including studies on twins and genotypic analyses. While genes may play an essential role in shaping eating attitudes, it is also important to consider environmental factors that may modify them. Findings indicate that food neophobia is the result of an interaction between genetics and the environment, highlighting the complexity of the phenomenon.

Research also confirms the link between sensory problems and food neophobia and ARFID (Avoidant/Restrictive Food Intake Disorder). Hypersensitivity to food’s texture, taste, smell, and appearance plays a key role in these disorders. Sensory sensitivity influences food preference and avoidance of certain foods, leading to a restricted diet and difficulty adapting to new foods. Much of this research has focused on how sensitivity to food’s texture, taste, smell, and appearance affects eating attitudes and behaviors. Sensory sensitivity has been strongly associated with avoidance of certain textures and tastes, resulting in reduced adoption of new foods [51]. It has been shown that individuals with higher sensory sensitivity were more likely to avoid foods with intense odors and unfamiliar appearances [48]. In a study by Norris M et al., participants showed strong avoidance of certain foods due to negative sensory experiences [53]. A study by Kauer J et al. demonstrated the role of disgust and sensory hypersensitivity in ARFID. People with ARFID often experience intense sensory reactions, leading to avoidance of certain foods. Disgust induced by food texture and smell was a key factor in food avoidance [43]. This is also confirmed by other studies [54,55,56].

Childhood experiences have a significant impact on the development of eating habits, including food neophobia (fear of trying new foods) and ARFID. Factors such as upbringing style, food-related trauma, and early interactions with food can influence attitudes toward food in later life. Children who experienced excessive parental control were more likely to avoid new foods. An upbringing style characterized by encouragement to try new foods positively led to less neophobia [57]. Regular exposure of children to various foods in early childhood was associated with less tendency toward neophobia in later life [58].

Cultural norms around food and childhood experiences, such as the availability of a variety of foods, had a significant impact on levels of neophobia in adulthood. It was indicated that children from families with lower socioeconomic status were more prone to neophobia [15]. Children who had experienced traumatic food-related events had a higher risk of developing ARFID, showing avoidance of specific foods or food-related situations. The results suggest that negative experiences with food, such as choking or allergic reactions, may lead to ARFID [59]. Early eating patterns, including picky eating, may lead to persistent eating disorders, such as ARFID, in later life. This study analyzed the trajectories of fussy eating in childhood and their impact on ARFID. It indicated that children with persistent picky eating were more likely to develop ARFID [60]. Family eating habits, such as excessive control or lack of variety in the diet, had a significant impact on the development of ARFID in children. It has been shown that children with ARFID often come from families with specific dietary patterns that influence their attitudes toward food [61]. Eating neophobia and ARFID are strongly linked to childhood experiences. Parenting, early experiences with food, trauma, and family eating patterns play a crucial role in shaping eating attitudes. Research shows that children who experience positive interactions with food and various culinary experiences are less likely to develop these disorders. Understanding these factors is key to developing effective therapeutic strategies and support for those with food neophobia and ARFID.

Understanding the interaction between childhood experiences, sensory sensitivities, genetics, environmental factors, eating neophobia, and ARFID is essential to developing effective prevention and treatment strategies. Through parental education, therapeutic support, and research into risk factors, it is possible to reduce the prevalence of these disorders and improve the quality of life of those affected. Further research into the links between childhood experiences, sensory sensitivity, and genetics may lead to a better understanding of the mechanisms behind food neophobia and ARFID. Creating intervention programs that combine parental education early in the onset of the disorder with therapeutic support can effectively reduce the disorder’s incidence, which will impact the later occurrence of the disorder in adulthood. Educational programs should be available to families of different income levels to ensure equal access to knowledge and support. Raising awareness about the impact of childhood experiences on eating disorders is critical. Parents should be educated to promote diversity and openness in eating and to avoid over-controlling children’s eating habits. Promoting positive food experiences and introducing children to different tastes and textures in a controlled way can help reduce the risk of developing neophobia and ARFID.

Dealing with ARFID and food neophobia in adults requires a complex and individualized approach. This includes therapeutic interventions, dietary support, and interventions to help people understand and overcome specific food-related fears and barriers. Therapies such as cognitive behavioral therapy, exposure therapy, and dietary support can help reduce food-related anxiety and introduce a more balanced diet. Psychosocial support, including support groups and family involvement, is crucial in recovery. It is essential that the approach is tailored to the individual patient’s needs and that therapy is continued over the long term to ensure lasting change and improved quality of life [42,44].

## 5. Strengths of the Study and Limitations

Participation in the survey was completely anonymous and voluntary, which increased the honesty of responses and reduced the impact of socially desirable responses. The study used standardized FNS and NIAS questionnaires, which are well-recognized and validated in food neophobia and ARFID research and provide high reliability and accuracy of results. This method makes it possible to reach a wide range of participants, increasing the diversity of the research sample.

Although the snowball method allows many respondents to be reached, it can lead to problems with sample representativeness. The sample may be skewed if participants recruit friends with similar demographic characteristics or interests. Self-reporting by respondents can lead to reporting errors, especially for data on weight, height, and other subjective variables; however, this type of survey is commonly used. The cross-sectional survey limits the ability to draw conclusions about causality. Follow-up studies would be needed to understand better the dynamics of changes in eating behavior and their relationship to neophobia and ARFID.

The snowball method used in our study has limitations that need to be considered. The technique may lead to some bias in the sample selection as participants are recruited through existing contacts, which may limit the diversity of the sample and affect its representativeness. However, food selectivity, food neophobia, and ARFID are not commonly diagnosed. Therefore, the use of the snowball method did not significantly impact the selection of the study group. In addition, this method may not provide full equality in representing different demographic groups, which is a significant limitation when analyzing differences between groups. Nevertheless, in our study, the participation of more than 300 respondents allows the results to be considered reliable in the context of the purpose of the study, and the snowball effect proved effective in collecting an adequate amount of data.

The survey provided valuable information on food neophobia and ARFID in the adult population, using standardized tools and a diverse sample of respondents. The strengths of the survey, such as anonymity, voluntariness, and the use of recognized questionnaires, increase the reliability of the results. These results have the potential to significantly contribute to our understanding of food neophobia and ARFID. However, some limitations, including the recruitment methodology and lack of control over the conditions under which the questionnaires were completed, may affect the interpretation of the results.

## 6. Conclusions

Both women and men have a similar risk of ARFID, suggesting that gender is not the main differentiating factor. The highest ARFID risk in the underweight group may indicate an association between low body weight and eating disorders, although the differences are not statistically significant. Older age groups (57–69 years and 70–77 years) show a higher risk of ARFID, which may suggest that age influences the development or severity of eating disorders. The higher risk of ARFID in people living in rural areas may be related to differences in food availability and variety. People with lower education (vocational and primary) show a higher risk of ARFID, which may indicate a need for nutrition education in these groups.

People with a higher risk of food neophobia avoid more foods, are less likely to eat foods prepared by a third party, are less open to introducing new ingredients into their favorite foods, and experience more negative emotions associated with eating unfamiliar foods. These findings suggest that food neophobia has a significant impact on various aspects of eating behavior and that its level can be linked to specific patterns of food avoidance and emotional responses to new foods.

The study shows a strong correlation between the risk of food neophobia and ARFID risk, indicating the need for further research and potential interventions in high-risk groups.

The results suggest the need for intervention-educational programs and psychological support for groups at higher risk of neophobia and ARFID, especially among older people, those with lower education, and those living in rural areas. Further research is needed to better understand the mechanisms leading to food neophobia and ARFID and to develop effective intervention strategies. Research should also include an analysis of the causes of these disorders in different demographic groups, allowing for a more personalized approach to their treatment and prevention.

## 7. Implications

### 7.1. Social Implications

There is a need for public education that raises awareness that eating disorders affect both sexes equally. The high risk of ARFID among the elderly and those living in rural areas highlights the importance of tailoring interventions to the specific needs of these groups. Social support, access to a variety of foods, and education about healthy eating may be vital in reducing the risk of ARFID and eating neophobia. Furthermore, the study highlights the importance of nutrition education in preventing health problems related to inadequate or inappropriate nutrition, especially among those with lower levels of education.

### 7.2. Management Implications

Healthcare management, particularly in the context of mental health and nutrition, requires the implementation of prevention and education programs targeting high-risk groups. The results suggest the need to integrate health and education services and to consider the diversity of access to food and specialized health and preventive care. Organizations such as the Ministry of Health, the Ministry of Education dealing with public health and health education, and medical institutions should consider introducing psychological support and nutrition education programs that are tailored to the needs of the elderly and less educated, e.g., in Poland running free Third Age Universities for seniors. Monitoring the effectiveness of these programs and making necessary changes based on the research results will be crucial for those managing them.

### 7.3. Policy Implications

From a policy point of view, the results of the study point to the need for public health strategies that consider the specific needs of different demographic groups, especially the elderly, those living in rural areas, and those with lower levels of education. Health policies should promote the availability of nutrition education and psychological support and develop intervention programs aimed at preventing ARFID and food neophobia. In addition, policymakers should consider regulations that increase the availability and diversity of food in rural areas, which can help reduce the risk of ARFID in these communities. Further state- or internationally-funded research is also needed to deepen the understanding of the mechanisms leading to the development of these disorders to enable more personalized and effective interventions.

## Figures and Tables

**Table 1:** Characteristics of the study group (n = 309).

	Values for the Test Group	
	n = 309	% = 100%
Gender:		
Woman	186	60.2
Male	123	39.8
Age (in years):		
18–30	140	45.3
31–43	76	24.6
44–56	64	20.7
57–69	22	7.1
70–77	7	2.3
Place of residence:		
Village	29	9.4
City of less than 20,000	40	13.0
City between 20,000 and 100,000	78	25.2
City over 100,000	162	52.4
Education:		
Primary school	1	0.3
Vocational Schools	41	13.3
High school	126	40.8
Bachelor’s degree	54	17.5
Master’s degree	87	28.2
Professional status:		
Unemployed	9	2.9
Working	188	60.8
Working pensioner	2	0.7
Working student	30	9.7
Student	54	175
Pensioner	26	8.5

**Table 2:** Risk of ARFID by subscale NIAS picky eating, appetite, and fear and risk of food neophobia (FNS).

Risk of ARFID			
NIAS Subscales	Cut-Off Point Indicating ARFID Risk	n Respondents n = 309	% of Respondents n = 309
NIAS–picky eating	>14 points	47	15.2
NIAS–appetite	>13 points	47	15.2
NIAS–fear	>14 points	34	11.0
**Risk of Food Neophobia**			
**Risk Assessment of** **Food Neophobia**	**Cut-Off Point Indicating** **the Risk of Food Neophobia**	**n Respondents** **n = 309**	**% of Respondents** **n = 309**
Low risk	<27 points	131	42.4
Medium risk	28–40 points	118	38.2
High risk	>41 points	60	19.4

**Table 3:** FNS food neophobia risk and the sum of NIAS subscales indicative of ARFID risk.

Risk of FNS Neophobia	Number of NIAS Subscales Indicative of ARFID Risk *								*p*-Value Chi2 Test
	0 Subscale Indicating ARFID Risk		1 Subscale Indicating ARFID Risk		2 Subscale Indicating ARFID Risk		3 Subscale Indicating ARFID Risk		
	n	%	n	%	n	%	n	%	
low risk of neophobia n = 131	119	90.84	11	8.40	1	0.76	0	0.00	*p* = 0.00231
medium risk of neophobia n = 118	88	74.58	26	22.03	4	3.39	0	0.00	
high risk of neophobia n = 60	17	28.33	19	31.67	10	16.67	14	23.33	
All respondents n = 309	224	72.49	56	18.12	15	4.85	14	4.53	

* ≥14 NIAS-avoidant eating, ≥13 NIAS-appetite, and ≥14 NIAS-fear.

**Table 4:** ARFID risk by gender, BMI, age, place of residence, education, and occupational status (n = 309).

	Average ± SD NIAS	Median * NIAS	Min–Max NIAS	*p*-Value Mann–Whitney U Test
Gender:				
Woman	25.5 ± 10.0	24	9–60	*p* = 0.943484
Male	25.1 ± 8.2	23	9–60	
All respondents	25.4 ± 9.3	23	9–51	
BMI categories:				
Underweight	30.7 ± 6.5	31.5	12–39	*p* = 0.776814
Normal body weight	24.9 ± 10.2	23	9–60	
Overweight	25.2 ± 8.6	23	9–51	
Obesity	25.2 ± 7.9	23.5	9–51	
Age (in years):				
18–30	24.2 ± 8.5	23	9–51	*p* = 0.147502
31–43	24.6 ± 9.7	22	9–51	
44–56	27.1 ± 10.2	25	9–60	
57–69	29.5 ± 9.3	26.5	18–51	
70–77	27.3 ± 5.9	29	18–34	
Place of residence:				
Village	29.1 ± 10.5	28	13–57	*p* = 0.739512
City of less than 20 000	25.9 ± 8.2	26	9–45	
City between 20,000 and 100,000	25.1 ± 10.2	22	9–52	
City over 100,000	24.7 ± 8.8	23	9–60	
Education:				
Vocation school	29.3 ± 8.1	29	17–46	*p* = 0.235516
Primary school	29 ± 0	29	29	
High school	25.4 ± 8.9	23	9–57	
Bachelor’s degree	25.5 ± 9.7	23	9–51	
Master’s degree	24.1 ± 9.8	22	9–60	
Professional status:				
Unemployed	32 ± 13.8	29	14–57	*p* = 0.893850
Working	24.7 ± 8.3	23	9–52	
Working pensioner	17 ± 7.1	17	12–22	
Working Student	24.7 ± 10.7	23	11–51	
Student	24.3 ± 9.4	23	9–51	
Pensioner	31.3 ± 10.7	29.5	18–60	
FNS				
Low risk	19.7 ± 5.7	20	9–37	*p* = 0.000000
Medium risk	25.8 ± 6.7	26	10–44	
High risk	36.6 ± 9.5	35	18–60	

* Because of the non-normal distribution of the above parameters, the appropriate measure of the mean is the median t.

**Table 5:** Food neophobia risk by gender, BMI, age, place of residence, education, and occupational status (n = 309).

FNS Scale Score				*p*-Value Mann–Whitney U test
	Average ± SD FNS	Median * FNS	Min–Max FNS	
Gender:				
Woman	32.1 ± 10.3	32	10–61	*p* = 0.019482
Male	30.6 ± 11.3	27	10–64	
All respondents	31.5 ± 10.7	30	10–64	
BMI categories:				
Underweight	38.0 ± 11.2	34	28–63	*p* = 0.403560
Normal body weight	30.86 ± 10.8	29	10–63	
Overweight	30.9 ± 9.8	29	10–58	
Obesity	32.5 ± 11.5	30	13–64	
Age (in years):				
18–30	29.3 ± 9.2	28.5	10–63	*p* = 0.337740
31–43	31.6 ± 12.0	29	10–63	
44–56	34.0 ± 10.8	33	10–64	
57–69	37.09 ± 12.1	35.5	20–58	
70–77	32.8 ± 6.7	34	22–40	
Place of residence:				
Village	33.8 ± 12.7	29	13–63	*p* = 0.650839
City of less than 20 000	30.2 ± 11.7	24	18–57	
City between 20,000 and 100,000	32.1 ± 10.7	30	11–64	
City over 100,000	31.0 ± 10.1	30	10–61	
Education:				
Vocation school	34.5 ± 12.0	32	13–64	*p* = 0.773930
Primery school	40.0	40	40	
High school	31.3 ± 10.1	29	10–63	
Bachelor’s degree	29.4 ± 9.8	27.5	10–57	
Master’s degree	31.6 ± 11.2	30	10–61	
Professional status:				
Unemployed	37.6 ± 10.0	39	18–55	*p* = 0.563368
Working	30.9 ± 10.6	29	10–64	
Working pensioner	24.0 ± 1.4	24	23–25	
Working Student	29.9 ± 10.4	27.5	16–58	
Student	29.7 ± 9.7	28	10–63	
Pensioner	40.3 ± 11.2	40	22–61	

* Because of the non-normal distribution of the above parameters, the appropriate measure of the mean is the median t.

**Table 6:** Relationship between level of risk of food neophobia and different aspects of eating behaviors.

		Low Risk of Neophobia		Medium Risk of Neophobia		High Risk of Neophobia		*p*-Value Chi2
		n = 131	%	n = 118	%	n = 60	%	
The number of foods that respondents do not eat	Some	110	83.97	78	66.10	35	58.33	*p* = 0.0325
	Several	17	12.98	35	29.66	19	31.67	
	Dozens	4	3.05	5	4.24	6	10.00	
Willingness to eat food prepared by a third party	Not	5	3.82	8	6.78	21	35.00	*p* = 0.0063
	I have no opinion	4	3.05	5	4.24	2	3.33	
	Yes	122	93.13	105	88.98	37	61.67	
Has your willingness to eat your favorite food enhanced with a product you have never eaten before?	Not	10	7.63	11	9.32	34	56.67	*p* = 0.000
	I have no opinion	0	0.00	7	5.93	3	5.00	
	Yes	121	92.37	100	84.75	23	38.33	
The sensation evoked by the consumption of a product/food that respondents were not familiar with	I have no problem eating a new meal or product	101	77.10	54	45.76	8	13.33	*p* = 0.000
	Uncertainty	14	10.69	36	30.51	20	33.33	
	Disgust	5	3.82	5	4.24	17	28.33	
	Anxiety	0	0.00	4	3.39	1	1.67	
	Horror (e.g., at the appearance. smell of a particular food)	10	7.63	10	8.47	4	6.67	
	Stress	1	0.76	9	7.63	10	16.67	

## Data Availability

The data presented in this study are available on request from the corresponding author. The data are not publicly available due to restrictions that apply to the availability of these data.
